# Editorial: Biomechanical performance and relevant mechanism of physical medicine and rehabilitation for neuromusculoskeletal disorders, volume II: aiding injury prevention and improving rehabilitation with a better understanding of relevant biomechanical mechanisms

**DOI:** 10.3389/fphys.2025.1719779

**Published:** 2025-10-23

**Authors:** Qi Wang, Qipeng Song, Pui Wah Kong, Cui Zhang, Dan Wang, Li Li

**Affiliations:** ^1^ Sport Science School, Beijing Sport University, Beijing, China; ^2^ Department of Sports and Health Science, Shandong Sport University, Jinan, China; ^3^ Physical Education and Sports Science Department, National Institute of Education, Nanyang Technological University, Singapore, Singapore; ^4^ Sports Biomechanics Lab, Shandong Institute of Sport Science, Jinan, China; ^5^ School of Physical Education and Sport Training, Shanghai University of Sport, Shanghai, China; ^6^ Department of Health Sciences and Kinesiology, Georgia Southern University, Statesboro, GA, United States

**Keywords:** rehabilitation, injury potential, lower extremity injury, neurological disorders, chronicdisorders

## Introduction

Neuromusculoskeletal disorders, such as sports-related injuries, e.g., chronic ankle instability (CAI), anterior cruciate ligament (ACL) injuries or patellofemoral pain (PFP); neurological disorders, e.g., cerebral palsy or stroke; and chronic disorders, e.g., knee osteoarthritis (KOA) or chronic obstructive pulmonary disease (COPD); are debilitating conditions that can lead to substantial functional impairment or even permanent disability. Despite its significant impact and ongoing burden at both personal and societal levels, the underlying mechanisms of physical medicine and rehabilitation remain poorly understood. The limited understanding in this field may impede the development of clinical rehabilitation strategies.

Biomechanical assessment may help better understand the mechanisms underlying physical medicine’s effectiveness and rehabilitation for neuromusculoskeletal disorders. From advanced infrared motion capture systems to finite element models, various modalities are available to investigate the changes in the physical function of individuals with neuromusculoskeletal disorders and to examine the differences in their physical function compared to uninjured individuals. Therefore, this Research Topic aimed to gather recent advancements to enhance the understanding of biomechanical performance and relevant mechanisms of physical medicine and rehabilitation for neuromusculoskeletal disorders. Sixteen articles have passed the peer review and were finally published. Among these papers, 10 works explained sports-related injuries, four articles explored neurological disorders, and two works discussed chronic disorders.

## Mechanisms of sports-related injuries

Preventing sports-related lower limb injuries remains a key area of research interest, with the primary objective of minimizing injury risk and elucidating the underlying mechanisms. Zou and their group investigate the effects of fatigue and anticipation on biomechanical risk factors of ACL injury during 180° pivot turns in female soccer players (Zou et al., 2024). They observed that fatigue and unanticipated tasks negatively impacted the lower-limb biomechanics and diminished movement performance among female soccer players, thereby increasing the risk of ACL injury during pivot turns. The authors recommended that cognitive training should be strengthened in female soccer players to mitigate the risk of non-contact ACL injuries under unanticipated scenarios during competitions. Xue and coworkers recruited 50 male participants, including those who had undergone ACL reconstruction on either the dominant (ACLR-D) or nondominant (ACLR-ND) limb, as well as healthy controls, and compared the biomechanical characteristics of bilateral limbs among these groups during a drop vertical jump task (Xue et al.). It was observed that compared to the surgical limbs, the nonsurgical limbs of ACLR patients may suffer an increased risk of ACL injury due to altered landing mechanics and neuromuscular control strategies. Moreover, the nonsurgical limb in the ACLR-ND group may be at greater risk than that in the ACLR-D group. These reports indicate that the impact of limb dominance should be considered in the rehabilitation of ACLR patients for better return to sport.

Within sports-related injuries, PFP is also one of the most prevalent conditions and differs from ACL injuries in that it is a nontraumatic knee problem. Nagahori and Ho focused on the relationship between patellar cartilage thickness and knee external rotation during squatting in individuals with and without PFP (Nagahori and Ho). They observed that thinner patellar cartilage was associated with increased knee external rotation during bilateral squatting, regardless of PFP status. These observations suggested that increased knee external rotation during squatting may negatively affect patellar cartilage thickness. The authors recommended that clinicians carefully assess KER during squatting in patients with or at risk of PFP. In addition to alterations in lower extremity kinematics, central nervous system adaptations have also been observed in individuals with PFP. Ho and colleagues investigated the central activation ratio of the gluteal muscles in individuals with and without PFP (Ho et al.). Their results suggested a tendency for individuals with PFP to exhibit a lower gluteus maximus central activation ratio, although the difference was not statistically significant. Furthermore, a higher gluteal central activation ratio was associated with better function in this population. The above studies provide valuable insights for optimizing rehabilitation strategies in individuals with PFP.

Investigating the biomechanical characteristics of individuals with CAI during landing from a height and strategies for reducing the potential risk of ankle sprains remains a primary focus in rehabilitation research. Compared to individuals without CAI, Zhong and colleagues reported that those with CAI demonstrate a greater potential risk of injury, as indicated by greater ankle inversion angles and angular velocities during drop landings (Zhong et al.). Their study further revealed that, compared to single-task conditions, the ankle inversion angles and angular velocities under dual-task conditions differed among individuals without CAI. In contrast, no significant changes were observed in individuals with CAI (Zhong et al.). These reports suggest that individuals with CAI may exhibit maladaptive neuroplastic changes at the spinal and cortical levels, which could increase their potential risk of injury under dual-task conditions. Wang’s group investigated the biomechanical characteristics of the lower limbs during single-leg drop landings in individuals with unilateral functional ankle instability under various attention focus strategies (Wang et al.). They observed that external focus strategies promote a conservative landing strategy, while internal focus may enhance lower limb stability. Integrating these strategies into functional ankle instability rehabilitation programs can help reduce the risk of reinjury. The finite element method, by simulating the mechanical responses of the ankle’s lateral ligament, provides an effective means to evaluate the risk of ankle injuries (Zhu et al., 2025). Zhou et al. employed a finite element method to investigate alterations in neural control and stress response distribution during landing in patients with ankle ligament injuries (Zhou et al.)The results showed that the anterior talofibular and calcaneofibular ligaments’ laxity increases stress on the metatarsals. This ligament laxity may also trigger muscle compensation, which can further affect ankle joint stability and increase the risk of ankle injury.

The biomechanical changes in the lower extremity caused by CAI are not limited to the ankle joint but also affect the proximal knee and hip joints, thereby increasing the risk of injury (Zhang et al., 2024). Based on retrospective studies, He et al. reported that patients with CAI exhibit changes in biomechanical parameters during landing that are associated with an increased risk of ACL injury (He et al.). These changes include increased hip and knee extension moments, reduced hip flexion angles, elevated peak vertical ground reaction forces, and greater trunk lateral flexion angles. These reports provide a valuable basis for designing targeted prevention measures and rehabilitation programs to help reduce the risk of ACL injuries in individuals with CAI.

Running is a common form of exercise, but improper technique may contribute to sports-related injuries. Verdel et al. recruited 15 injury-free female runners to examine the effects of running speed, incline, and fatigue on the calcaneus angle (Verdel et al.). They observed that higher running speeds and fatigue conditions may elevate injury risk by increasing the range of motion. In contrast, inclined running may reduce this risk by limiting excessive eversion and range of motion. These reports provide useful insights for developing injury prevention strategies relevant to runners and researchers.

## Rehabilitation strategies for sports-related injuries

Musculoskeletal injuries can lead to significant functional deficits mediated by the central nervous system (CNS), including increased arthrogenic muscle inhibition and altered neuroplasticity (Dong et al., 2024). In sports-related injury rehabilitation, addressing CNS dysfunction is essential for optimal recovery. Huang et al. utilized transcranial direct current stimulation (tDCS) and Bosu intervention on the injury potential during drop landing in people with CAI (Huang et al.). They reported that, compared to the Bosu intervention alone, transcranial direct current stimulation combined with the Bosu intervention was more effective in reducing peak ankle inversion angular velocity, plantarflexion angle at the moment of peak ankle inversion, and advancing time to peak ankle inversion, and that both interventions reduced the peak ankle inversion angle. Their reports provide new insights into the clinical development of rehabilitation strategies for individuals with CAI. They indicate that integrating CNS-directed interventions, such as transcranial direct current stimulation, into conventional functional training can help reduce the risk of recurrent ankle injuries.

## Mechanisms and rehabilitation strategies of neurological disorders

A common neurodevelopmental disorder, Cerebral Palsy (CP), leads to significant and lasting impairments of the neuromuscular system. Pontiff et al. investigated the relationships of the Power Leg Press performance with walking capacity and self-reported performance and participation in ambulatory individuals with CP to understand how muscle power influences activity capacity and participation (Pontiff et al.). Their observations demonstrated a significant positive association between leg press power, walking capacity, and self-reported walking performance and mobility-related participation in ambulatory individuals with CP. These results suggest that clinicians should incorporate lower extremity power into their assessments of individuals with CP and recommend using the Power Leg Press test. In another work, 10 participants with typical development and 8 participants with CP were included (Damiano et al.). The results showed that after the body weight supported treadmill training, the CP group exhibited beneficial effects on kinematics, which supports the basic premise for applying such interventions in neurorehabilitation at the body structure level.

Neurodegenerative disorders are emerging as major public health challenges. Stroke is one of the leading causes of adult death worldwide and can cause severe movement dysfunction and limitations, such as walking difficulties. Liu et al. explored immediate changes in temporal and spatial parameters of gait and the joint angles in people suffering from stroke throughout the entire gait cycle after the application of the lower extremity elastic strap binding technique (Liu et al.). This report stated that for people suffering from stroke, the lower extremity elastic strap binding technique can help reduce the hip and knee flexion limitations and decrease the ankle plantarflexion and inversion angles during walking. These benefits potentially result from proprioceptive feedback that stimulates changes in the excitability of the motor cortex to promote effective coordinated movement (Song et al., 2021). This offers a complementary rehabilitation strategy for improving gait in people who have suffered a stroke with foot drop and limited hip and knee flexion.

In another study, Yang et al. recruited ten individuals with stroke to assess spatiotemporal gait parameters and symmetries immediately after split-belt treadmill training and single-belt treadmill training, as well as after a 5-minute rest following each intervention (Yang et al.). The study concluded that single-belt and split-belt treadmill training effectively improved gait speed and step length on the shorter side in patients with stroke. Furthermore, the results showed improvement in step length symmetry immediately after split-belt treadmill training, without impairing other temporal symmetries. However, this effect diminished after a 5-minute rest. This work highlights the potential of split-belt treadmill training to enhance gait symmetry. It may provide valuable insights for developing more efficient and safer gait rehabilitation strategies for patients with stroke.

## Rehabilitation strategies for chronic disorders

The pain associated with KOA has increased the likelihood of tripping over obstacles. Pain-relieving transcutaneous electrical nerve stimulation (TENS) is widely utilized in treating KOA; however, it exhibits limited analgesic effects and modest benefits for functional ability. On that basis, a total of 23 participants with KOA were randomized to either the tDCS + TENS group or the TENS-only group to evaluate the effects of the two interventions on pain reduction and gait optimization during obstacle crossing among older adults with KOA (Zhang et al.). The study concluded that the combination of tDCS and TENS was significantly more effective than TENS alone in reducing pain and improving gait adaptability during obstacle negotiation among older adults with KOA. These reports offer new perspectives to guide the development of future rehabilitation strategies for individuals with knee osteoarthritis.

Among older adults, COPD is prevalent and exerts substantial impacts on quality of life, morbidity, and mortality. Vocal therapy enhances respiratory muscle strength and endurance, with singing training emerging as an increasingly popular pulmonary rehabilitation program for patients with COPD. Qiao et al. investigated the content of vocalization training for patients with COPD by examining differences in respiratory muscle activation across various vocalization tasks (Qiao et al.). The study observed that vocal loudness, rather than pitch or vowels, should be the key factor in voice training for these patients. This research provides clinical significance for implementing voice training.

This Research Topic presents the latest approaches to physical medicine and rehabilitation interventions for neuromusculoskeletal disorders. It encourages the use of biomechanical approaches to evaluate the effectiveness and explore the mechanisms of these interventions. We hope this work will inspire scholars researching biomechanical performance and relevant mechanisms of physical medicine and rehabilitation for neuromusculoskeletal disorders to explore these research frontiers further.
